# Damp Buildings: Associated Fungi and How to Find Them

**DOI:** 10.3390/jof10020108

**Published:** 2024-01-27

**Authors:** Evangelia Loukou, Nickolaj Feldt Jensen, Lasse Rohde, Birgitte Andersen

**Affiliations:** 1 Division of Building Technology, Management and Indoor Environment, Department of the Built Environment, Aalborg University, A.C. Meyers Vænge 15, DK-2450 Copenhagen, Denmark; elou@build.aau.dk (E.L.); nfj@build.aau.dk (N.F.J.); 2 Division of Energy and Sustainability in Buildings, Department of the Built Environment, Aalborg University, Thomas Manns Vej 23, DK-9220 Aalborg, Denmark; lro@build.aau.dk

**Keywords:** fungal growth, building materials, indoor mycobiota, water damage, moisture problems, sampling techniques, detection methods, identification methods, minimum water activity

## Abstract

The number of buildings experiencing humidity problems and fungal growth appears to be increasing as energy-saving measures and changes in construction practices and climate become more common. Determining the cause of the problem and documenting the type and extent of fungal growth are complex processes involving both building physics and indoor mycology. New detection and identification methods have been introduced, and new fungal species have been added to the list of building-related fungi. However, the lack of standardised procedures and general knowledge hampers the effort to resolve the problems and advocate for an effective renovation plan. This review provides a framework for building inspections on current sampling methods and detection techniques for building-related fungi. The review also contains tables with fungal species that have been identified on commonly used building materials in Europe and North America (e.g., gypsum wallboard, oriented strand board (OSB), concrete and mineral wool). The most reported building-associated fungi across all materials are *Penicillium chrysogenum* and *Aspergillus versicolor*. *Chaetomium globosum* is common on all organic materials, whereas *Aspergillus niger* is common on all inorganic materials.

## 1. Introduction

Prolonged indoor exposure, prevalent in industrialised countries, significantly impacts the comfort, health and well-being of individuals [1]. The growth of fungi and bacteria in the humid or wet built environment is one of the key issues of indoor air contamination [2,3] and plays an essential role in occupational and public health problems [4,5]. Indoor mould has been associated with adverse health effects [2,4,6,7,8]. Some indoor fungal species are responsible for exacerbation of asthma [9], and a recent study suggests that fungal cell wall components, proteins and enzymes can significantly affect respiratory health [4].

Increased humidity is the most critical factor for indoor fungal growth [2,3,10,11]. Fungal growth in damp buildings is a common problem due to condensation on interior surfaces, water damage from burst pipes or flooding. Furthermore, increased indoor humidity can cause damage to the building construction and materials [12,13] and triggers chemical emissions [14]. The World Health Organization (WHO) has estimated that 10–50% of homes in Europe, North America, Australia, India and Japan face moisture-related problems [2].

Even though fungal spores are ubiquitous, not all fungal species can grow everywhere [15]. Buildings constitute new habitats for fungi to grow and proliferate [4,16,17,18]. These artificial, inorganic environments have different characteristics than natural habitats that fungi have occupied for millions of years [17]. Therefore, the fungal biodiversity indoors is distinct and limited compared to the natural habitats they originate from. While fungal spores can be introduced indoors from various sources such as the soil, food products, potted plants, pets and humans [19], as well as from building materials themselves [20,21], the predominant and primary source is the outdoor air [22].

Not all building materials are equally susceptible to fungal growth. The characteristics of the building material and its moisture content determine which species can grow on it [4] and which mycotoxins and other metabolites will be produced and released into the indoor environment [23,24]. The composition and availability of organic compounds are also critical factors for the suitability of materials to serve as a nutrient source [25]. Consequently, different materials are prone to be colonised by specific fungal species, contingent on the fortuitous deposition of the fungal spores on the designated material and the alignment of moisture level and nutrient composition with the fungus’ needs [4,15,20,26].

The demand to increase energy savings in the built environment has led to new construction techniques and increased airtightness of the building envelope. However, if these measures are not properly designed and implemented, there is a high risk of moisture increase indoors, condensation on the internal surfaces and thus, fungal growth [27]. The combination of highly insulated external walls and inadequate ventilation due to faulty design, installation, operation or maintenance is the main reason for fungal contamination in low-energy buildings [28,29].

Furthermore, fungal contamination of buildings has also a socioeconomic aspect, as it is connected to poor housing conditions, fuel poverty and energy crises [30]. Fungal problems are more common and severe in low-income communities due to the lack of maintenance, insulation, ventilation and heating of buildings [2]. Additionally, indoor space overcrowding leads to increased moisture production that does not correspond to the original mechanical ventilation rates [31]. Lately, due to the energy crisis of 2022, many European countries have recommended lowering indoor temperature by 1 or 2 degrees, which might seem minor. However, it may result in colder interior surfaces and, therefore, increases the risk of condensation and fungal growth.

Sampling, detecting and identifying fungi are important aspects of controlling and preventing fungal growth in the built environment when water damage has occurred. There exists a broad variety of sampling techniques and detection methods but no specific procedures, guidelines or standards for how they should be carried out. Therefore, the results of a building inspection are often not reproducible. Different inspectors may reach different conclusions on the severity, extent and remediation measures for the fungal growth and the building. Each sampling technique has advantages and limitations, while factors like the sampling location significantly affect the outcome of the analysis [18].

Detection of fungal growth in a moisture-damaged building without species identification is not sufficient to address and solve the problem effectively. Different fungal species have different requirements even though they belong to the same fungal genus. Genera like *Alternaria*, *Aspergillus* and *Penicillium* are commonly encountered in the indoor air and dust, but different species have different origins. Some species are food-borne (baseline spora) (e.g., *Penicillium digitatum* on citrus fruit), whereas others are associated with building materials (indicator species) (e.g., *P. chrysogenum* on wallpaper) [15,18,32,33]. In addition, species’ identity can aid in locating the contamination source and choosing the best detection and removal strategy [15,24,34,35]. Fungal identity can also reveal potential health effects associated with species-specific exposure and potential mycotoxin production [4,36,37,38,39].

However, the information on fungal species’ identity presented by the WHO [2], which is still the most authoritative reference, is outdated, and so are the recommended fungal strains in the various ISO standards for sampling, identification and material testing [40,41,42,43,44,45,46,47]. Lastly, simulation programs for heat and moisture transport and prediction of fungal growth in building constructions also need revision on the relevant fungal species and their moisture requirements.

This review is addressed to researchers, health professionals, legislators, international organisations for standards, building physicists and building inspectors. The purpose of this review is (1) to introduce an inventory containing the most common indoor fungal species in northern Europe and America, the building materials with which they are associated and their minimum water activity for indoor growth and (2) to provide a framework for building inspections on current sampling and detection methods for indoor fungal growth.

## 2. Requirements for Fungal Growth

Fungi’s life cycle includes three phases: (1) spore germination, (2) mycelium growth and (3) spore formation (sporulation). During the first two phases, vegetative growth takes place, while the third phase consists of the fungus’ reproduction [11]. When the environmental conditions are right, the fungal spores that have settled on the different surfaces start germinating. A mycelium is produced, a multi-cellular filamentous structure, to allow food intake. Fungi secrete extracellular enzymes and acids, which break down the growth medium/substrate to access the nutrients they need [35]. During this process, particles, gases and microbial volatile organic compounds (MVOCs) are released into the environment. After the mycelium has grown enough, spores are created from the fruiting bodies, while the mycelium continues growing to produce more spores and ensure further spreading of the microorganism in its habitat. As nutrient availability decreases, the fungus’ life is endangered, and so sporulation increases to ensure its survival and further propagation [11]. Thus, spore diffusion is relatively independent of the growth conditions.

Fungi are resilient colonisers that can germinate and grow on most natural and man-made materials. Studies have shown that fungal growth can begin after just a short period of favourable conditions, while the spores can survive for a long time after the contaminated materials dry and the growth ceases [11,20,25,48]. Several requirements affect the appearance of fungal contamination, including the extent and rate of growth, as well as the produced metabolites. Even though the factors are interdependent and interconnected, they could be classified into three groups for clarification purposes. The most important of them are further analysed in continuation.

1. Abiotic factors for fungal growth:
  - temperature [11,35,49,50],
  - moisture [11,13,26,35,48,49,50,51,52,53],
  - nutrients [11,13,26,35,50,51,52],
  - UV radiation [54];
2. Composition and properties of the building material [11,13,16,26,35,49]:
  - moisture capacity and moisture transport properties [11,26,54],
  - organic nutrient content [11,13,26,35,50,51,52,55],
  - chemical environment [11,16,26,35,50,51,56];
3. Characteristics of the fungal species:
  - preferences and colonising potential [49],
  - interactions between the fungal species and other microorganisms [11].

### 2.1. Abiotic Factors for Fungal Growth

#### 2.1.1. Temperature

Fungi can tolerate a wide range of temperatures, from 0 to 50 °C [11,54]. However, their optimal temperature range for growth is narrower, as fungi enter a dormant state at low temperatures of 0–5 °C by slowing down their metabolic activities, while most fungal species die at high temperatures above 46 °C [35]. Most building-related fungal species have a temperature optimum between 20 and 25 °C [32], which coincidentally is also the desired temperature range in buildings for thermal comfort.

#### 2.1.2. Moisture Content, Water Activity or Relative Humidity

Several factors describe the state of water in materials, i.e., water activity, osmotic pressure, fugacity, water potential and water content [57]. As fungi mostly grow on surfaces, they utilise unbound, available water on the surface of the substrate (i.e., the building material), not what is trapped inside it [57,58]. The water activity $\left(a_{w}\right)$ of surfaces can be used to directly assess the moisture availability for fungal growth [57,59]. The material’s moisture content is another useful factor, while the air relative humidity (RH) only affects the moisture level indirectly [48]. The $a_{w}$ refers to the ratio of the vapour pressure of pure water in the material to the vapour pressure of pure water at the same conditions of temperature and pressure [22] $(a_{w}$ × 100 = % RH at equilibrium) [33]. Every fungus has specific moisture requirements, meaning it has a minimum, a maximum and an optimum $a_{w}$ for growth. Although the minimum $a_{w}$ may differ from species to species, the optimum level typically ranges between 0.90 and 0.99 [22,60]. Generally, an $a_{w}$ of 0.75 in a material is considered critical for fungal growth [54]. Nevertheless, a study published by Bastholm et al. [34] found that certain fungal species could grow in museum repositories under controlled RH levels of below 60% ($a_{w}$ = 0.60). Frazer et al. [61] showed that $a_{w}$ and temperature directly influence the germination, growth and sporulation of *Stachybotrys chartarum*. These findings are also supported by Ayerst [60], Grant et al. [62] and Johansson et al. [63], who deduced that an increase in temperature and nutrient availability leads to a lower requirement of $a_{w}$ for fungal growth.

Often, the fungal growth rate (mm/d) or germination time (d) is plotted as a function of temperature and relative humidity/water activity (isopleth systems). These graphs are species-specific, based on the fungus’ growth requirements. Isopleths provide useful information on the influence of environmental conditions on the growth of fungi. However, they are developed under well-defined, steady-state conditions, which is rarely the case in practice. In buildings, the environmental conditions are highly transient, including long-term, yearly fluctuations (seasonal) and short-term, daily variations mainly due to the users’ activities [10].

The growing medium has an influence, mainly due to its $a_{w}$, on species detection and enumeration. Furthermore, the use of different standard media can serve for species identification, as the fungal colonies/conidia colour is determined by the media composition and added trace metals [32]. Finally, the different media are complemented with antibiotics to suppress the contamination of the cultures from bacteria [32]. Some of the most-used media covering a wide water activity range are the following: V8 juice agar (V8), Malt Extract Agar (MEA), Dichloran-Glycerol agar (DG18), Malt Yeast agar with 40 or 50% Glucose (MY40G or MY50G). The recipes for these media according to Samson et al. [32], Hocking and Pit [64,65] and Simmons [66] are provided as a Supplementary Material File. Two media can be used (e.g., DG18 together with V8 or MEA) to cover most building-related fungi [53]. In special cases, e.g., archives and museum repositories, media with very low $a_{w}$ can be used [34], like MY40G or MY50G [32].

Based on their moisture requirements, fungi are divided into groups: hydrophilic, mesophilic and xerophilic [26]; the grouping into primary, secondary and tertiary colonisers [48,62,67] has become obsolete since water damage is not necessarily a progression. Between mesophilic and xerophilic fungi, a group for xerotolerant fungi can be added [68]. It is well documented that the most important factor dictating fungal growth on building materials is the moisture availability [3,11,35,53,54,57,69], as dust and dirt that can serve as nutrient sources usually are present in all houses [2,14]. Table 1 summarises the minimum water activity requirements of some building-related fungal species under normal indoor environmental conditions (22 °C ± 3 °C), along with their media preferences.

Furthermore, the duration of moisture exposure or time-of-wetness (TOW) [10] and the time of wet–dry cycles under fluctuating conditions are also important factors. Pasanen et al. [69,70] examined the spores’ behaviour under fluctuating conditions of temperature and relative humidity and suggested the hypothesis that conidia may be able to adapt to an unstable environment to survive [70]. Additionally, it has been observed that the spores’ viability can be lost after fast drying conditions in cases where they have adjusted to high moisture levels [70,71,72]. The contamination rate under fluctuating condensation incidents is much slower than fast-appearing contamination in events of capillary water absorption by materials during flooding or other water damage incidents [70].

**Table 1:** Minimum water activity $\left(a_{w}\right)$ requirements of representative fungal species.

	$a_{w}$ [References]	Genus	Species	Media [References]
**Hydrophilic**	0.95 [56,73]	*Acremonium*	*charticola*	V8 [73]; MEA [74]
	0.94–0.95 [22,61,73]	*Stachybotrys*	*chartarum*	V8 [15]; MEA [32]
	0.94 [73,74]	*Chaetomium*	*globosum*	V8 [15]; MEA [32]
	0.92 [73,74]	*Rhodotorula*	*mucilaginosa*	MEA [32]
	0.90–0.95 [73,74,75]	*Trichoderma*	*viride*	V8 [73]; MEA [74]
**Mesophilic**	0.89–0.90 [62,74]	*Alternaria*	*chartarum*	V8 [15]
	0.86–0.91 [22,74]	*Epicoccum*	*nigrum*	V8 [15]
	0.85–0.89 [22,62,73,74]	*Alternaria*	*alternata*	V8 [73]; DG18 [32]
	0.85–0.88 [22,73,76]	*Cladosporium*	*herbarum*	V8 [15]; MEA, DG18 [32]
	0.84–0.87 [22,62,73,76]	*Cladosporium*	*cladosporioides*	V8 [15]; MEA, DG18 [32]
	0.82–0.85 [22,60,73]	*Aspergillus*	*fumigatus*	MEA, DG18 [32]
	0.82–0.84 [62,73,74,76]	*Cladosporium*	*sphaerospermum*	V8 [15]; MEA, DG18 [32]
	0.80 [74]	*Penicillium*	*corylophilum*	MEA, DG18 [32]
**Xerotolerant**	0.79–0.80 [22,74]	*Paecilomyces*	*variotii*	MEA, DG18 [32]
	0.78–0.85 [22,62,73,74,75]	*Penicillium*	*chrysogenum*	MEA, DG18 [32]
	0.77–0.78 [60,73,74]	*Aspergillus*	*niger*	V8 [34]; MEA, DG18 [32]
	0.74–0.79 [22,62,73,77]	*Aspergillus*	*versicolor*	V8 [15]; MEA, DG18 [32]
	0.78 [22,74]	*Aspergillus*	*sydowii*	MEA, DG18 [32]
**Xerophilic**	0.69 [22,73,74]	*Wallemia*	*sebi*	DG18, MY50G [32]
	0.68 [78]	*Aspergillus*	*halophilicus*	MY50G [34]
	0.59 [79]	*Aspergillus*	*penicillioides*	DG18, M40, M40Y [32,80]

**Figure 1:**
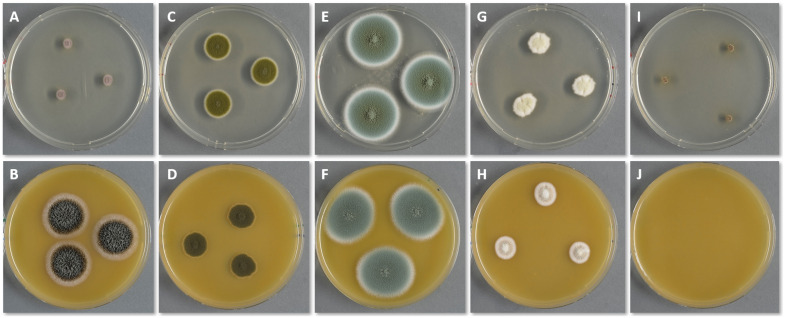
Pure cultures of indoor fungi on DG18 (top row) and V8 (bottom row). *S. chartarum* (**A**,**B**), being hydrophilic, grows poorly on DG18. *C. herbarum* (**C**,**D**), which is mesophilic, as well as *P. chrysogenum* (**E**,**F**) and *A. versicolor* (**G**,**H**) that are xerotolerant, grow well on both media. Conversely, the xerophilic *W. sebi* (**I**,**J**) does not grow on V8.

Figure 1 shows the growth of pure cultures on two different media (DG18 on the top row and V8 on the bottom) after 7 days of incubation at 20 °C. *S. chartarum* (A and B), *C. herbarum* (C and D), *P. chrysogenum* (E and F), *A. versicolor* (G and H), *W. sebi* (I and J).

### 2.2. Composition and Properties of Building Materials

The characteristics of the building material serving as substrate play an essential role in the appearance of fungal growth and species diversity. The material’s surface structure, hygroscopicity, porosity, water permeability, etc., directly affect moisture availability. Different materials have varying moisture sorption capacity [13]. For instance, plywood, OSB and gypsum board are hygroscopic, meaning they tend to absorb moisture, thereby increasing their susceptibility to fungal growth [16,55,81]. In contrast, glass, ceramic products, polymer-based materials, etc., are hydrophobic and thus more mould-resistant [16,26,82]. Furthermore, it has been shown that the required RH for porous materials is higher than 80%, as water does not become readily available on the surface [35]. On the other hand, some materials cannot support growth under conditions of high moisture content, and proliferation initiates when they start drying out [13,52]. Conversely, the results of Vanpachtenbeke et al. [81] suggest that a liquid water source might be necessary for initiating fungal growth on wood materials.

The composition of building materials determines the nutrient availability on its surface, which is a key driver for the material’s susceptibility to fungal growth and abundance. When the environmental conditions are favourable for fungal germination, fungi diffuse enzymes into the substrate in order to break down the required nutrients, which can be used for their growth [18]. Building materials have distinct compositions and contain different organic compounds, which can be a good nutrient source for most fungi or just for specific species that can utilise them. Such components can be low molecular weight carbohydrates (e.g., glucose, dextrose), free sugars, natural organic polymers (e.g., starch, pectin, cellulose, hemicellulose, lignin, etc.) or other readily accessible nutrients [16,22,55]. For example, materials rich in organic matter, e.g., wood, plywood, the paper layer of gypsum board, ceiling tiles, etc., are especially good substrates due to their complex polymers [13,16,35,52,83]. On the other hand, paper-free materials or materials with lime composition (e.g., inorganic ceiling tiles, gypsum, etc.) are less susceptible to mould formation [16,52,84]. Furthermore, when processing or treating materials, their properties are being altered. For example, solid wood contains cellulose layers, which are connected with lignin; fungi colonising solid wood need to be able to break down both of these components. However, some of these components are removed during wood processing, while chemicals and glues may be added. During the pulping or chipping of wood, the pH and chemical characteristics change, and the crushing of cells results in cellulose break, lignin removal and release of sugars and starches [18]. Therefore, different materials can serve as food for particular fungal species. Even different parts of the same wood species do not share the same properties; for example, sapwood is high in free sugar content, in contrast to heartwood, which is more mould resistant [55]. Additionally, specific molecules isolated from wood extractives have antifungal activity [85]. Still, the extractives tend to vary based on the wood species, its geographical origin and the part of the tree [16,86]. Finally, material additives like oily coating, wax supplements, glues and adhesives (e.g., starch adhesive in the paper layers of gypsum board, phenol-formaldehyde in OBS) can aid or inhibit fungal formation and growth [55,84].

Finally, the material’s chemical composition affects the microorganisms’ growing environment. The salinity, alkalinity, oxygen content and pH influence the environment’s quality and control whether the organisms can germinate. Jensen et al. [25] demonstrated that even though high pH levels will prevent fungal growth, *A. versicolor* spores can survive harsh pH conditions during extended periods until a more favourable environment occurs and they can germinate again.

### 2.3. Characteristics of Building-Associated Fungal Species

Fungi have developed mechanisms that allow them to access their necessary nutrients [85]. The several different fungal species on a material interact with each other, which can create mutually beneficial or competitive relations. The growing fungi metabolise the components of the material and produce new nutrients that become available for other microbial organisms to use and proliferate in succession [18]. On the other hand, some species can produce toxins to inhibit other organisms (e.g., metabolites against bacteria) with the same growing requirements so they can claim the material [18]. The resulting metabolic products like allergens and toxins are related to the components and nutrients provided by the substrate and the species acting as colonisers [4,19,37,38,67,87,88]. Furthermore, synergistic effects have also been reported between fungi and bacteria, like the case of *Serpula lacrymans*, a wood-colonising fungal species [89].

## 3. Associated Fungi of Common Building Materials

Several researchers have studied the connections between different building materials and fungal genera or species [20,26,33,90,91,92,93] or the relation between fungi and their preferred growing conditions [34,63,75,82,94,95]. Andersen et al. [33] have shown that *P. chrysogenum* and *A. versicolor* are the most frequently found fungal species in buildings with water damage. At the same time, their study shows a particular connection between specific fungal species and building materials, which is also supported by Hyvärinen et al. [26]. For example, research suggests that the introduction of gypsum drywall as a construction material after the 1940s has introduced new fungal species in buildings [96]. At the same time, there is evidence that gypsum wallboard is often pre-contaminated by specific fungal species from the production stage, before even reaching the construction site [20]. Another study has associated *C. globosum* with OBS [21]. Consequently, different materials are prone to be colonised by specific species. Even when examining the same material, its different components and layers can support the growth of different species, i.e., *A. versicolor* on the liners of plasterboard, *S. chartarum* and *Penicillium spinulosum* on the core [97].

The tables present the most reported species, with their current/new name. Within the last 10 years, fungal taxonomy has undergone a major revision, and some of the common building-related species have changed names [15]. For example, all *Ulocladium* species have moved to the genus *Alternaria*. Table 2 lists the name changes of building-related fungal species as recorded in the Index Fungorum [98] and Mycobank Database [99].

The building materials collected in this review have been selected based on their widespread usage in the building industry. Only papers where the type of building material was unambiguous have been included. The materials are grouped according to (1) their composition, which dictates the availability, quality and quantity of nutrients; (2) the processing level and (3) their use. The resulting groups are the following:

- Massive wood and woodchip materials (Table 3).
- Gypsum board/drywall, paper/cardboard and wallpaper (Table 4).
- Inorganic materials: paint, plaster, concrete and fibreglass wallpaper (Table 5).
- Insulation materials: bio-based, foam-based and mineral-based (Table 6).

In the cases of older studies conducted by the same researchers, only the latest publication has been included. For example, species reported in the earliest publications by Samson, Flannigan and Adan from 1994 and 2011 [22,57,100,101] have not been included in the tables, as the associations are reported by Samson et al. (2019) [32]. The same applies to former publications by Nielsen [67] and Schmidt [102]. In addition, papers reporting associations between building materials and fungal genera [26,90,91,92,93,103] have not been included either.

**Table 2:** Name changes of building-related fungal species according to the Index Fungorum.

Old Name	Current Name
*Acremonium furcatum*	*Furcasterigmium furcatum*
*Acremonium kiliense*	*Sarocladium kiliense*
*Acremonium strictum*	*Sarocladium strictum*
*Antrodia vaillantii*	*Fibroporia vaillantii*
*Arthrinium phaeospermum*	*Apiospora sphaerosperma*
*Aspergillus ornatus*	*Sclerocleista ornata*
*Chaetomium murorum*	*Botryotrichum murorum*
*Cryptococcus albidus*	*Naganishia albida*
*Engyodontium album*	*Parengyodontium album*
*Epicoccum purpurascens*	*Epicoccum nigrum*
*Geomyces pannorum*	*Pseudogymnoascus pannorum*
*Lecanicillium kalimantanense*	*Gamszarea kalimantanensis*
*Monocillium tenue*	*Niesslia tenuis*
*Mucor globosus*	*Mucor racemosus*
*Mucor spinosus*	*Mucor plumbeus*
*Paecilomyces lilacinus*	*Purpureocillium lilacinum*
*Penicillium purpurogenum*	*Talaromyces purpureogenus*
*Penicillium variabile*	*Talaromyces wortmannii*
*Phellinus contiguus*	*Fuscoporia contigua*
*Phoma glomerata*	*Didymella glomerata*
*Poria placenta*	*Rhodonia placenta*
*Rhodotorula minuta*	*Cystobasidium minutum*
*Rhodotorula rubra*	*Rhodotorula mucilaginosa*
*Scopulariopsis brevicaulis*	*Microascus brevicaulis*
*Scopulariopsis fusca*	*Scopulariopsis asperula*
*Ulocladium atrum*	*Alternaria atra*
*Ulocladium botrytis*	*Alternaria botrytis*
*Ulocladium chartarum*	*Alternaria chartarum*
*Verticillium lecanii*	*Akanthomyces lecani*
*Verticillium luteoalbum*	*Acrostalagmus luteoalbus*
*Verticillium nigrescens*	*Gibellulopsis nigrescen*

### 3.1. Wood and Woodchip Materials

The first group of building materials consists of wood, either massive or composed of different sizes of woodchips and different levels of processing. Massive wood is a natural material comprising structural polymers, i.e., cellulose fibres, hemicellulose and lignin, and non-structural constituents called extractives. In contrast, woodchip-containing materials are engineered products manufactured by bonding woodchips by using adhesives (such as resins or glues) or compressed under heat and pressure. Materials like particleboard, OSB, medium-density fibre (MDF), chipboard, plywood, Masonite board, etc., are included in this subcategory.

The majority of fungal species encountered indoors belong to the phyla of *Ascomycota* and *Mucoromycota* [32]. However, some species belonging to *Basidiomycota* are important wood and timber decay fungi [32,104] but are not part of the table. The most commonly reported of these species are the following:
*Amyloporia xantha* [105]*Gloeophyllum sepiarium* [104,105]*Antrodia sinuosa* [104,106,107]*Gloeophyllum trabeum* [104]*Asterostroma cervicolor* [104]*Neoantrodia serialis* [106]*Coniophora marmorata* [104]*Phlebiopsis gigantea* [106]*Coniophora puteana* [104,105,106,107]*Rhodonia placenta* [107]*Donkioporia expansa* [104,105,107]*Serpula himantioides* [104,106]*Fibroporia vaillantii* [104,106,107]*Serpula lacrymans* [104,105,106,107,108]*Fuscoporia contigua* [104]*Tapinella panuoides* [104,106]*Gloeophyllum abietinum* [104,105]


Table 3 shows that species of *Alternaria, Chaetomium, Cladosporium, Penicillium, Stachybotrys* and *Trichoderma* are common on all wood materials, while *Aspergillus* species dominate on woodchip materials.

**Table 3:** Fungal species on wood and wood-fibre materials.

Material Type	Genus	Species	References
Common for all	*Alternaria*	*alternata*	[18,32,109]
	*Alternaria*	*chartarum*	[18,53,109]
	*Alternaria*	*tenuissima*	[32,33]
	*Aspergillus*	*creber*	[15,18]
	*Aspergillus*	*versicolor*	[15,18,53,110,111]
	*Apiospora*	*sphaerosperma*	[33]
	*Aureobasidium*	*pullulans*	[18,33]
	*Chaetomium*	*globosum*	[15,18,32,53,110]
	*Cladosporium*	*dominicanum*	[15]
	*Cladosporium*	*halotolerans*	[15]
	*Cladosporium*	*herbarum*	[32,33,53,109]
	*Cladosporium*	*langeronii*	[111]
	*Cladosporium*	*sphaerospermum*	[15,18,32,33,53,109]
	*Microascus*	*brevicaulis*	[18,32]
	*Oidiodendron*	*griseum*	[18,32]
	*Paecilomyces*	*variotii*	[18,33]
	*Penicillium*	*aurantiogriseum*	[18]
	*Penicillium*	*brevicompactum*	[18,53,109]
	*Penicillium*	*commune*	[18]
	*Penicillium*	*chrysogenum*	[15,18,53,111]
	*Penicillium*	*corylophilum*	[53,111]
	*Penicillium*	*decumbens*	[18]
	*Rhodotorula*	*mucilaginosa*	[33]
	*Sarocladium*	*strictum*	[18,32]
	*Stachybotrys*	*chartarum*	[18,87,93,103,110]
	*Talaromyces*	*flavus*	[18]
	*Trichoderma*	*atroviride*	[53,111]
	*Trichoderma*	*harzianum*	[18,32,53,109]
Wood-fibre materials	*Aspergillus*	*amstelodami*	[18]
	*Aspergillus*	*glaucus*	[18,110]
	*Aspergillus*	*nidulans*	[18]
	*Aspergillus*	*ochraceus*	[18]
	*Aspergillus*	*repens*	[18]
	*Aspergillus*	*sydowii*	[18,53]
	*Aspergillus*	*ustus*	[18]
	*Chaetomium*	*cochlioides*	[32]
	*Chaetomium*	*elatum*	[32]
	*Epicoccum*	*nigrum*	[111]
	*Geotrichum*	*candidum*	[18]
	*Microascus*	*melanosporus*	[32]
	*Naganishia*	*albida*	[112]
	*Penicillium*	*simplicissimum*	[18]
	*Talaromyces*	*purpurogenus*	[18]
Massive wood	*Alternaria*	*atra*	[53]
	*Aspergillus*	*niger*	[33,110]
	*Aureobasidium*	*melanogenum*	[32]
	*Cephalotrichum*	*gorgonifer*	[32]
	*Cephalotrichum*	*microsporum*	[32]
	*Cladosporium*	*allicinum*	[15,32]
	*Cladosporium*	*cladosporioides*	[32,109]
	*Cladosporium*	*macrocarpum*	[32]
	*Cladosporium*	*variabile*	[109]
	*Coniochaeta*	*hoffmannii*	[32]
	*Coniophora*	*puteana*	[83,110]
	*Didymella*	*glomerata*	[18,110]
	*Furcasterigmium*	*furcatum*	[112]
	*Fusarium*	*equiseti*	[109]
	*Geomyces*	*pannorum*	[113]
	*Penicillium*	*dierckxii*	[18]
	*Penicillium*	*expansum*	[53]
	*Penicillium*	*palitans*	[32,53]
	*Penicillium*	*roqueforti*	[32]
	*Penicillium*	*spinulosum*	[18]
	*Penicillium*	*thomii*	[18]
	*Pleurostoma*	*richardsiae*	[32]
	*Pseudogymnoascus*	*pannorum*	[32]
	*Rhodotorula*	*mucilaginosa*	[53]
	*Sistotrema*	*brinkmanii*	[18]
	*Talaromyces*	*wortmannii*	[32]
	*Trichoderma*	*citrinoviride*	[53]
	*Trichoderma*	*koningii*	[18]
	*Trichoderma*	*longibrachiatum*	[32,53]

### 3.2. Gypsum Board, Paper/Cardboard and Wallpaper

The second group also contains wood-based materials, in which the wood has been heavily processed, and consists of wood fibres. Gypsum board has a core of gypsum, which is a naturally occurring mineral composed of calcium sulfate dihydrate and paper finishes on both sides. Due to the paper, gypsum board, together with acoustic and ceiling tiles that have a similar composition, are grouped as organic materials. Paper and cardboard are listed together, as they are both manufactured from processed wood pulp. Even though wallpaper can be made from various materials such as paper, fabric or vinyl, this table specifically addresses wallpaper derived from wood pulp. Wood pulp is produced by mechanically or chemically breaking down cellulose fibres, which are then formed into sheets. The key differences between them lie in thickness, layering, surface treatment or the use of certain additives to enhance specific properties depending on their intended use.

Table 4 shows that species of *Aspergillus, Chaetomium, Penicillium* and *Stachybotrys* are common on all paper/cardboard materials, *Alternaria* species are found on gypsum and wallpaper, while *W. sebi* is found on other paper/cardboard materials.

**Table 4:** Fungal species on gypsum board, paper/cardboard (drywall, ceiling tiles, acoustic tiles) and wallpaper.

Material Type	Genus	Species	References
Common for all	*Aspergillus*	*sydowii*	[18,32,53]
	*Aspergillus*	*versicolor*	[15,18,26,32,33,38,53,93,110,111,112,114,115,116]
	*Chaetomium*	*cochlioides*	[32]
	*Chaetomium*	*elatum*	[32]
	*Chaetomium*	*globosum*	[15,18,32,53,110,114,117]
	*Cladosporium*	*cladosporioides*	[111,115]
	*Microascus*	*brevicaulis*	[32,110]
	*Penicillium*	*chrysogenum*	[15,18,20,32,33,38,53,111,114,115]
	*Penicillium*	*rubens*	[15,18,32]
	*Stachybotrys*	*chartarum*	[15,18,20,32,38,53,87,91,93,110,112,116]
	*Stachybotrys*	*chlorohalonata*	[32]
Common for gypsum board and wallpaper	*Alternaria*	*alternariae*	[15,32]
	*Alternaria*	*alternata*	[18,32]
	*Alternaria*	*atra*	[53]
	*Alternaria*	*chartarum*	[18,38,53,115]
	*Alternaria*	*tenuissima*	[32,53]
	*Aspergillus*	*niger*	[38,114,115]
	*Aspergillus*	*ustus*	[18,38,114]
	*Cladosporium*	*sphaerospermum*	[15,18,32,53,115,117]
	*Penicillium*	*aurantiogriseum*	[18,115]
	*Penicillium*	*brevicompactum*	[18,32]
	*Penicillium*	*commune*	[18,38]
	*Penicillium*	*corylophilum*	[18,32,38,53,111]
	*Penicillium*	*glabrum*	[18,38,114]
	*Penicillium*	*simplicissimum*	[18,115]
	*Penicillium*	*verrucosum*	[38,114]
	*Sarocladium*	*strictum*	[18,32]
Common for paper/cardboard and wallpaper	*Scopulariopsis*	*asperula*	[32,38]
	*Trichoderma*	*atroviride*	[38,103]
	*Wallemia*	*sebi*	[103,110]
Common for paper/cardboard and gypsum board	*Aspergillus*	*hiratsukae*	[15,32]
Paper/cardboard	*Aspergillus*	*fischeri*	[32]
	*Aspergillus*	*spinosus*	[32]
	*Curvularia*	*geniculata*	[32]
	*Didymella*	*glomerata*	[32]
	*Epicoccum*	*nigrum*	[32]
	*Geotrichum*	*candidum*	[32]
	*Memnoniella*	*echinata*	[32]
	*Myxotrichum*	*chartarum*	[113]
	*Niesslia*	*heterophora*	[110]
	*Oidiodendron*	*griseum*	[32]
	*Scopulariopsis*	*candida*	[32]
	*Trichoderma*	*koningii*	[103]
	*Trichoderma*	*viride*	[103]
Gypsum board	*Ascotricha*	*chartarum*	[113]
	*Aspergillus*	*creber*	[15,18]
	*Aspergillus*	*glaucus*	[18]
	*Aspergillus*	*nidulans*	[18,114]
	*Aspergillus*	*ruber*	[18]
	*Aureobasidium*	*pullulans*	[18]
	*Botryotrichum*	*murorum*	[15]
	*Candida*	*parapsilosis*	[15]
	*Cladosporium*	*halotolerans*	[32]
	*Cystobasidium*	*minutum*	[112]
	*Gibellulopsis*	*nigrescens*	[15]
	*Memnoniella*	*echinata*	[18,32]
	*Microascus*	*brevicaulis*	[18]
	*Paecilomyces*	*variotii*	[18]
	*Penicillium*	*citrinum*	[18]
	*Penicillium*	*decumbens*	[18]
	*Penicillium*	*dierckxii*	[18]
	*Penicillium*	*spinulosum*	[18]
	*Rhodotorula*	*mucilaginosa*	[112]
	*Talaromyces*	*purpurogenus*	[18]
	*Talaromyces*	*variabilis*	[18]
	*Trichoderma*	*harzianum*	[18]
Wallpaper	*Alternaria*	*botrytis*	[111]
	*Aspergillus*	*fumigatus*	[115]
	*Cladosporium*	*herbarum*	[32,53]
	*Penicillium*	*carneum*	[38]
	*Penicillium*	*crustosum*	[38]
	*Penicillium*	*digitatum*	[115]
	*Penicillium*	*italicum*	[38]
	*Penicillium*	*olsonii*	[38]

### 3.3. Paint, Plaster, Concrete and Fibreglass Wallpaper

The third group includes inorganic materials with different primary components and distinct applications. Paint is a mixture of pigments, binders, solvents and additives for surface decoration. Plaster is composed of materials like gypsum, lime or cement and has usually a high pH value. Concrete is made with cement, water and aggregates to construct building elements. Finally, fibreglass wallpaper consists of woven fibreglass strands coated with a resinous binder for reinforcement or decoration of interior wall surfaces.

Table 5 shows that species of *A. versicolor, A. niger* and *P. chrysogenum* are common on all inorganic materials, while *Cladosporium* and *Wallemia* species dominate on plaster and paint. Concrete and fibreglass wallpaper have, compared to other materials, only a limited number of associated species.

**Table 5:** Fungal species on inorganic materials: paint, plaster, concrete and fibreglass wallpaper.

Material Type	Genus	Species	References
Common for all	*Aspergillus*	*niger*	[32,33,115,116]
	*Aspergillus*	*versicolor*	[15,33,53,56,115]
	*Penicillium*	*chrysogenum*	[15,32,33,53,56,115]
Common for paint and plaster	*Acremonium*	*charticola*	[15,56]
	*Alternaria*	*alternata*	[115,116]
	*Alternaria*	*chartarum*	[115,116]
	*Cladosporium*	*cladosporioides*	[115,116]
	*Cladosporium*	*dominicanum*	[15]
	*Cladosporium*	*halotolerans*	[15,32]
	*Cladosporium*	*herbarum*	[32,53]
	*Cladosporium*	*sphaerospermum*	[15,32,33,53]
	*Paecilomyces*	*variotii*	[115]
	*Penicillium*	*corylophilum*	[32,53]
	*Stachybotrys*	*chartarum*	[87]
	*Wallemia*	*muriae*	[15]
	*Wallemia*	*sebi*	[15,32]
Common for paint and concrete	*Penicillium*	*brevicompactum*	[32,116,118]
Paint	*Akanthomyces*	*lecanii*	[15]
	*Aspergillus*	*canadensis*	[15]
	*Aspergillus*	*fumigatus*	[115]
	*Aspergillus*	*ustus*	[115]
	*Aureobasidium*	*pullulans*	[53]
	*Cladosporium*	*allicinum*	[15]
	*Debaryomyces*	*hansenii*	[15]
	*Didymella*	*glomerata*	[32]
	*Epicoccum*	*nigrum*	[22]
	*Niesslia*	*tenuis*	[15]
	*Penicillium*	*aurantiogriseum*	[115]
	*Penicillium*	*roseopurpureum*	[15]
	*Penicillium*	*simplicissimum*	[115]
	*Penicillium*	*viridicatum*	[115]
	*Rhodotorula*	*mucilaginosa*	[53]
	*Scopulariopsis*	*candida*	[115]
Plaster	*Acrostalagmus*	*luteoalbus*	[32]
	*Alternaria*	*alternariae*	[32]
	*Alternaria*	*tenuissima*	[32]
	*Aspergillus*	*flavus*	[56]
	*Aspergillus*	*westerdijkiae*	[32]
	*Cephalotrichum*	*gorgonifer*	[32]
	*Cladosporium*	*allicinum*	[15]
	*Cladosporium*	*langeronii*	[56]
	*Furcasterigmium*	*furcatum*	[56]
	*Gamszarea*	*kalimantanense*	[56]
	*Microascus*	*melanosporus*	[32]
	*Mortierella*	*alpina*	[56]
	*Mucor*	*racemosus*	[116]
	*Parengyodontium*	*album*	[56]
	*Purpureocillium*	*lilacinum*	[56]
	*Sarocladium*	*kiliense*	[56]
	*Verticillium*	*zaregamsianum*	[56]
	*Wallemia*	*ichthyophaga*	[15]
Concrete	*Aspergillus*	*fumigatus*	[33,118]
	*Aspergillus*	*melleus*	[33]
	*Aspergillus*	*niger*	[32,33]
	*Aspergillus*	*ochraceus*	[33]
	*Aspergillus*	*westerdijkiae*	[32]
	*Didymella*	*glomerata*	[32]
	*Mucor*	*racemosus*	[33]
	*Mucor*	*plumbeus*	[33]
Fibreglass wallpaper	*Aureobasidium*	*pullulans*	[33]

### 3.4. Insulation Materials: Bio-Based, Foam-Based, Mineral-Based

The last group contains insulation materials with different compositions. Bio-based insulation is made from renewable, organic resources. Foam-based insulation contains polymers and chemicals, which result in lightweight, rigid or flexible materials (e.g., Polyurethane, Polyisocyanurate, Polystyrene, Polyethylene, etc.). Mineral-based insulation materials are derived from naturally occurring minerals (e.g., Rockwool, Fibreglass, etc.). Each type of these materials has unique properties and is suitable for specific applications.

Table 6 shows that inorganic insulation materials, like other inorganic materials, have *A. niger, A. versicolor* and *P. chrysogenum* as the dominating species.

**Table 6:** Fungal species on insulation materials: bio-based, foam-based and mineral-based.

Material Type	Genus	Species	References
Common for all	*Aspergillus*	*niger*	[111,119,120,121]
	*Aspergillus*	*versicolor*	[53,93,120]
	*Cladosporium*	*cladosporioides*	[119,120,121]
	*Penicillium*	*chrysogenum*	[53,111,120,121]
	*Stachybotrys*	*chartarum*	[120,121]
Common for bio- and mineral-based	*Alternaria*	*tenuissima*	[32,119]
	*Alternaria*	*chartarum*	[119]
	*Cladosporium*	*herbarum*	[119]
	*Cladosporium*	*sphaerospermum*	[32,119]
	*Epicoccum*	*nigrum*	[119]
Common for foam- and mineral-based	*Paecilomyces*	*variotii*	[121]
	*Penicillium*	*corylophilum*	[53,111,121]
	*Trichoderma*	*viride*	[119,121]
Bio-based insulation	*Alternaria*	*botrytis*	[120]
	*Aspergillus*	*amstelodami*	[120]
	*Aspergillus*	*flavus*	[120]
	*Talaromyces*	*wortmannii*	[120]
Foam-based insulation	*Alternaria*	*alternata*	[121]
	*Alternaria*	*botrytis*	[121]
	*Amorphotheca*	*resinae*	[121]
	*Curvularia*	*lunata*	[122]
	*Epicoccum*	*nigrum*	[121]
	*Penicillium*	*aurantiogriseum*	[121]
	*Penicillium*	*decumbens*	[121]
	*Penicillium*	*glabrum*	[121]
	*Penicillium*	*janthinellum*	[122]
	*Sclerocleista*	*ornata*	[121]
	*Talaromyces*	*purpurogenus*	[121]
	*Trichoderma*	*harzianum*	[121]
Mineral-based insulation	*Cladosporium*	*allicinum*	[32]
	*Cladosporium*	*langeronii*	[111]
	*Trichoderma*	*atroviride*	[111]
	*Trichoderma*	*pseudokoningii*	[119]

### 3.5. The Building-Associated Fungal Species

In this review, 132 fungal species from 51 genera are reported for humid or water-damaged buildings. From these, only two species, *A. versicolor* and *P. chrysogenum*, could be found on all material types.

Materials, partly or totally organic, could support the growth of 102 different species, while on the inorganic materials, 70 different species were found. A total of 40 species were common to both organic and inorganic materials, with species like *A. alternata, C. sphaerospermum, P. variotii* and *P. corylophilum* being the most reported. Other species like *A. glaucus* and *C. globosum* were only found on organic materials, while *A. charticola* and *M. spinosus* only on inorganic materials.

## 4. Building Evaluation Process

Visible fungal growth on interior surfaces, furniture and other household effects is the most common reason for starting an investigation. However, often, an investigation is launched even in the absence of visible fungal growth because the occupants or building users experience mouldy odours and/or adverse health effects. An investigation can also be initiated before the renovation of a water-damaged building, e.g., due to flooding or another water-damage incident. Regardless, high humidity or water ingress is always the reason for the presence of fungal growth even though the source of water is not obvious or the building has dried out.

The purpose of an inspection is to ascertain the existence of fungal growth, to locate the source of humidity/water and to design a remediation plan. Knowing which fungal species are growing on a particular material and the preferred $a_{w}$ of the fungal species can ensure that all fungal growth is discovered and the correct renovation strategy is proposed [18].

**Figure 2:**
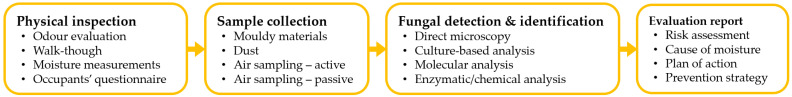
Fungal contamination assessment process of damp buildings.

To assess the building-related fungal contamination risk and confirm any moisture problems, it is necessary to quantify the fungal load, identify the microbial diversity and determine the contamination source. The assessment procedure is performed in four phases: (1) physical inspection of the building, (2) sample collection, (3) fungal detection and identification and (4) evaluation report. Figure 2 depicts this process and potential steps.

Fungal growth can be seen in buildings as discolouration, stains or blots on walls, floors and ceilings, especially on colder surfaces like thermal bridges, below windows, behind furniture, etc. When fungal growth is visible, the procedure is straightforward: to clean off/demolish the affected area, restore it and perform quality control. However, fungal growth can also be hidden in the building construction, cavities and behind the wallpaper or sit in plain sight but be colourless, thin and patchy, thus easily overlooked.

All fungal growth, visible or unseen, can release equally high concentrations of fungal particles in the indoor environment [123], and it can be recognised through high humidity, musty odours or complaints of negative health symptoms by the occupants. Nonetheless, it can be challenging to find and sample, while restoration can be costly. Therefore, it is estimated that there is significant under-reporting of these cases. The microbial assessment of damp and mouldy buildings is an interdisciplinary challenge, spanning across the fields of mycology, building science and public health [124].

There are various sampling techniques that can be used for sample collection, while different detection methods can be applied to the collected samples. Some samples can be analysed by several detection methods, while others are intended for specific analysis. In the following sections, the sampling techniques and detection and identification methods are first analysed independently. Subsequently, it is described how the sampling techniques can be paired with the commercially used detection and identification methods.

### 4.1. Physical Inspection

A thorough walk-through inspection of the building is pivotal. Through the visual inspection, the investigator can reveal evidence of current or past water ingress, detect critical/problematic areas with humid or mouldy spots and evaluate the mouldy odour, which can be indirect evidence of hidden fungal growth. For that, investigators need to have a broad knowledge of moisture transport in buildings, material properties and behaviour and be able to identify the potential areas for increased or concealed humidity [18]. Usually, a walk-through inspection by experienced investigators can be sufficient to determine the cause and location of fungal growth and decide which analyses are required.

The inspection can be combined with a survey/questionnaire for the occupants about the experienced indoor air quality, possible symptoms or health problems related to fungal growth, their daily airing routines, cleaning practices, heating, ventilation and air conditioning (HVAC) system and the type and state of the building (e.g., past water damage incidents, insulation level, renovation works, etc.).

### 4.2. Sample Collection

With sampling, a specimen of fungal biomass is collected for subsequent qualitative or quantitative analysis in the laboratory [125] and assessment of the severity of fungal growth. The authors of this review suggest dividing the sampling techniques into three groups based on the sample origin: (1) material/surface sampling (spores, spore-producing structures and mycelium), for direct evidence; (2) dust collection (long-term sedimentation of spores) and (3) air sampling (snapshot of air-borne spores), for indirect evidence.

#### 4.2.1. Material Sampling

Sampling of visible fungal biomass directly where it grows is the first and most obvious choice to characterise the fungal contamination of a building. Material sampling techniques are normally used for genus or species identification. A surface sample can be taken to determine whether a stain is caused by fungal growth or another issue [126,127] or the effectiveness of remediation measures [37]. Additionally, material sampling can determine which fungal species originate from the building materials and not from an outdoor origin [128]. Nonetheless, the identified fungi are limited to those present in the specific area that is being sampled [126].

Smaller parts of building material/construction (bulk samples) or larger parts of surfaces (scrapings and shavings) can be removed for analyses in the laboratory [18]. Surfaces can also be sampled and tested for fungal growth using contact plates (for cultivation), sterile swabs (for cultivation or enzymatic analysis) [129] or tape lifts (for microscopy), which is relatively economical and quick [130]. The tape-lift method can be used to complement culture or enzymatic methods. For example, a tape-lift sample can be taken prior to a contact plate [33] or a swab [73]. On the other hand, the use of contact plates, swabs and tape lifts has been designed for smooth surfaces, which raises concerns about their performance on rough or porous materials [131,132]. Finally, contact plate and swab samples are quite sensitive to handling processes during retrieval (e.g., time and pressure applied on the contact plate, material of the swab, etc.) [131].

#### 4.2.2. Dust Sampling

Settled dust, 3 to 6 months old, can be a good proxy for either hidden fungal growth or for evaluation of long-term exposure of occupants to fungal particles. Dust sampling can be performed using sterile swabs, dust fall collectors (DFCs) or electrostatic dust fall collectors (EDCs) for long-term collection. Swab samples (usually 10 cm^2^) are obtained from horizontal surfaces 1.5 m or more above floor level on places that are not cleaned regularly, like on top of doors or picture frames, curtain rails, bookcases or cupboards [15]. As DFCs (usually 60–100 cm^2^), an empty, sterile Petri dish without medium can be used [124] or even a cardboard box with aluminium foil-covered inner surfaces [133]. Using the DFC method, airborne dust and fungal particles can be sampled over hours, days, weeks or even months, depending on the aim of the study [134]. EDCs [18,124,133,135] have been mostly used for exposure studies to endotoxins [135]. Floor dust, 1 to 3 weeks old, can also be sampled using a nozzle with micro-vacuum cassettes attached to an ordinary vacuum cleaner [127,136] or by analysing directly the dust collected from an ordinary vacuum cleaner bag (usually the whole living area) [137] and used to identify the present fungal particles [73,138]. When collecting dust samples, the choice of the sampling area, number of samples and order to take them when coming from the same surface need to be considered [15].

#### 4.2.3. Air Sampling

Air sampling provides a short-term exposure assessment through the collection of airborne fungal biomass. It can be done either passively (sampling over time) or actively (volumetric sampling). Passive air sampling is normally performed using Petri dishes containing growth medium, exposing the agar surface to the air for 30–60 min [38]. Active air sampling is carried out by using a device (sampler) drawing in a predefined volume of air. The most commonly used air sampling devices are (1) impactors and sieve samplers, (2) impingers, (3) filter samplers and (4) centrifugal and cyclonic samplers [18,125,139]. Impactors and sieve samplers collect a fixed volume of air impacted onto a Petri dish with growth medium or an adhesive surface (i.e., glass slides or membranes coated with a transparent, sticky substance). Centrifugal and cyclonic samplers use circular flow patterns to increase the airflow and deposit the airborne particles into a liquid, semi-solid or solid growth medium [125]. Impingers diffuse the collected air into a liquid medium, while filter samplers diffuse it into a sterile microporous filter [18].

Even though the sampler’s performance plays a role in the sampling quality [125], it is considered minor compared to the variability of microbe concentrations in the air [140]. A study conducted by Haas et al. [141] to compare the effectiveness of active impaction air sampling and passive sedimentation under standardised conditions showed that impaction can be more efficient, as it allows faster collection of a higher volume of air. These results are also supported by Thio et al. [142], whose study showed that large-volume air sampling can detect a wider spectrum of fungal species. Furthermore, it has been demonstrated that the indoor environment preparation prior to sampling (activated vs. non-activated testing) has an impact on the fungal biomass and species richness that can be detected since larger particles are susceptible to the sampling height and activation of the fungal reserves [143]. Non-activated air sampling can provide a representation of the situation at the specific time that the sampling takes place, while dust samples can describe a long-term exposure period.

#### 4.2.4. Choice of Sampling Techniques

**Figure 3:**
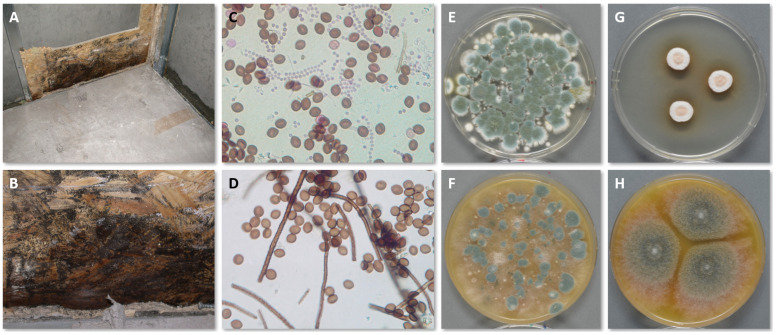
Growth of *C. globosum* in the interface between OSB and gypsum wallboard (**A**,**B**). Tape lifts from the OSB and direct microscopy mostly reveal *C. globosum* (**D**), but some *Penicillium* conidia in chains are also present (**C**). Active air sampling onto DG18 (**E**) and V8 (**F**) show mostly *Penicillium* spp. because the conidia of *C. globosum* do not become as airborne as *Penicillium* conidia. Pure cultures of *C. globosum* on DG18 (**G**) and V8 (**H**) also show its hydrophilic nature by better growing on V8 than DG18.

During the sampling process, several parameters and choices can affect the outcome of the investigation, and they must be considered in advance. In most cases, the purpose of the inspection dictates this decision-making process. Figure 3 shows the growth of *C. globosum* in the interface between OSB and gypsum wallboard (which has been removed) following a basement flood together with tape lifts, air samples on Petri dishes and pure cultures for identification.

The air sample volume determines the concentration of biomass that can be detected and is dictated by the sampling time and airflow rate [18,144,145]. The sampling time can vary greatly, from minutes to months, based on the selected sampling method and the needs of the investigation. The exact sampling location is important, especially for dust sampling, as the proximity to the source of fungal growth affects the concentration of spores [15]. Additionally, the spore distribution indoors (both in the air and dust) is random, spore liberation is sporadic and it is species-dependent if the spores can become air-borne [15]. Consequently, air sampling favours species that produce large quantities of small, dry spores (e.g., *Aspergillus, Cladosporium, Penicillium* spp.), while species that produce smaller amounts of large spores or spores in slime might be missed when sampled higher than 1.5 m from the floor (e.g., *Acremonium, Chaetomium, Stachybotrys, Trichoderma* spp.) [15]. Finally, data resulting from different methods, e.g., samples taken from the source and air samples [132] or under different conditions (i.e., different samplers, flow rates, sampling times and growth media [18]), cannot and should not be directly compared. The advantages and limitations of the above-mentioned sampling techniques are summarised in Table 7.

### 4.3. Fungal Detection and Identification

Detection and identification methods concern the laboratory analyses of the collected samples to confirm the presence of fungal contaminants, estimate the fungal load and/or perform species identification. The analysis can be quantitative, assessing the amount of fungal biomass, or qualitative, listing the identity of the different fungal species. Samples can be analysed using microscopy, cultivation or molecular methods for identification and chemical/enzymatic methods for biomass determination.

#### 4.3.1. Direct Microscopy

Using a dissecting or stereo microscope (×40 magnification), fungal growth can be observed directly on bulk materials, scrapings or shavings. For tape lifts, either directly from the fungal-infested materials or the bulk materials, scrapings or shavings, a light microscope (×400 magnification) is used. When performing microscopy analysis directly on the material, identification can typically be carried out to genus level only, while its use is limited in highly contaminated sites or samples due to overloading [147]. There is no need for an incubation period, and samples can be analysed directly, making this method low-cost and fast. On the other hand, there are no protocols and guidelines for the analysis, and identification demands a skilled mycologist. Therefore, it is not possible to standardise the processes between different laboratories [18].

#### 4.3.2. Culture-Based Analysis

Traditionally, the most-used method has been culture-based analysis. It can be applied to most sample sources and types, while it can be used for species identification. On the other hand, it is time- and labour-intensive and requires skilled mycologists for correct species identification. For culture analysis, spores, fungal fragments or microparticles are collected and cultivated in different media in the laboratory under controlled conditions. The media selection and growing conditions are of great importance to the outcome of the analysis. Each cultivation medium favours specific genera and species, and it is, therefore, necessary to use a variety of media (e.g., DG18, V8, MEA) to cover the whole spectrum of indoor fungi [53]. Even the selected technique to introduce the sampled organic matter in the Petri dish (e.g., scattering, shaking, direct or dilution plating) can influence the growth rate and detected species [18,148]. Consequently, it can be argued that culture-based methods may underestimate the taxonomic variety of present microorganisms [148,149].

#### 4.3.3. Molecular Analysis

Molecular analysis of fungal biomass by quantitative polymerase chain reaction (qPCR) or next-generation sequencing (NGS) has been gaining popularity in recent years, as it can provide quantitative results of high specificity, precision and sensitivity [103,129,147]. Culture-independent methods can detect both viable and most non-viable fungal fragments. The method has a fast analysis turnaround, and identification does not require highly trained mycologists. There are two approaches to molecular diagnostics, qPCR assays (commercial use) are designed to detect targeted, known species, while NGS (research use) provides higher discovery power to identify any species present [150].

In qPCR, the results are limited to selected species only, usually around 20 depending on the assay that is used commercially. In NGS, which is used for research purposes, the identification can be as good as the used repository [15], keeping in mind that for many organisms, a unique and identifiable genomic region has not been archived in databases yet [145,151]. There are some uncertainties concerning whether nucleic acids can be used as a representative measure for fungal biomass and if it is possible to recover the same molecular rates from all examined microorganisms, given the significant variability between fungal species [18]. Although the technology is still relatively new, there is promising potential for standardisation control among different analysts and laboratories [18]. A study by Adams et al. [124] showed that using a targeted approach to identify a fungal signature could make it possible to detect moisture damage in buildings.

#### 4.3.4. Enzymatic/Chemical Analysis

Finally, chemical tests use surrogate markers [132] detecting specific proteins, enzymes or other organic compounds. Usually, these tests provide an assessment of the indoor microbial load. A widely used commercial method for the built environment is the β-N-acetylhexosaminidase (NAHA) enzyme test, which assesses the indoor microbial load [152]. The test has been developed for both surface and air sampling and lies in the detection of the NAHA enzyme [143]. Other tests target compounds like adenosine triphosphate (ATP) or protein residues to determine the level of cleanliness of surfaces.

#### 4.3.5. Other Methods

There is a plethora of studies that have investigated the use of MVOCs and ergosterol, which could be used as biological markers [23,24,88,136,153]. For example, the particulate (1→3)-β-D-Glucan is a carbohydrate that has been extensively researched as a measure of fungal biomass [18,23,134,154]. However, no commercial methods are available yet for assessing indoor environmental contamination due to the difficulty of determining the emission source [136]. Recently, a different, non-traditional approach for hidden mould detection is using trained mould-detecting dogs [155]. Limulus amoebocyte lysate preparation (LAL test) and enzyme-linked immunosorbent assay (ELISA) targeting enzymes, proteins and other specific agents like allergens [18] are other available methods. However, the application of these techniques is outside the built environment (e.g., clinical studies, infection control, exposure assessment) or they have not been standardised to be commercially available yet [18,37,125] and therefore are beyond the scope of this review.

Table 8 summarises the different sampling techniques and corresponding detection methods for fungal growth assessment of buildings.

#### 4.3.6. Choice of Analytical Methods

All methods have strengths and weaknesses, and at present, no single method can be used to reliably confirm whether there is moisture or microbial damage [34,103,124,156]. Culture-dependent methods are highly selective due to the growing conditions, medium properties and the fact that heavy spore-producing, tolerant or general species are overestimated as they outgrow predominantly mycelial taxa and slow-developing, more fragile or specialised fungi [18,148]. In addition, spores’ viability is species-specific [18]. Molecular analysis can detect non-viable spores and fragments that cannot grow in a culture. That information can provide long-term insight, for example, about older water damage incidents that might have dried out or dead spores coated in toxins that might still be present, as spores and fragments can be allergenic despite their culturability [15,18]. On the other hand, culture methods do not require special equipment; they are widely used, well characterised and extensively researched and reliable reference data are available [147]. Even though molecular analysis can provide a high resolution, it can be costly, as it is only carried out at certified laboratories with specific and expensive equipment. PCR-based methods have not been validated enough yet for moisture damage incidents and health effects to be used for routine analyses [147], as the choice of genetic markers/genomic regions to be amplified influences the results of the analysis [145,157]. Additionally, molecular methods are sensitive to contamination, so care should be given when handling the samples [18]. Microscopy methods cover living fungi but also non-viable or dormant material, as well as structures that cannot be grown or contain no nuclei. On the other hand, the analysis process can be labour- and time-consuming, with a high risk of misidentification due to the analyst’s lack of skills or experience [18,147]. NAHA air and surface analysis can also detect the biomass of living and dead fungi and provide information about the presence of a fungal source. Still, it cannot characterise the present microbiological contaminants. Finally, there are more do-it-yourself tests, but they come with high uncertainty about the sampling process and knowledge level of the user. Table 9 recaps the advantages and limitations of the most commonly used detection methods.

Each company uses different methods to detect fungal growth, and often, scientific characterisation of the methods’ limitations and specifications is narrow or missing [3]. The investigations’ outcomes depend on many parameters, including the experience and knowledge level of the investigator and often, independent inspections result in different or contradicting conclusions that are not easily reproducible, making the problem even more complex. Examining the same area, or even the same fungi, can result in a different outcome, depending on the used approach [34], as there are no generally accepted methods and protocols [15,125]. Even though the topic is well documented and researchers have delved into the process of assessing microbial exposure and contamination [125,129,130,132,133,158,159], the need for evaluation and standardisation of the procedures is stressed [125,127,132,144]. There is a consensus that no method alone can give the whole picture, so a combination of techniques seems to be the best solution, as they complement each other [18,34,145,153,156], as all methods are biased in one way or another [125]. However, specific guidelines on which methods should be chosen and how they should be used are still lacking. There is a need for comparison, evaluation and validation of the different techniques and methods commonly used for sampling, detection and identification of indoor microbial pollution and the development of clear guidelines.

### 4.4. Evaluation Report

After the completion of the investigation, the outcome of the inquiry needs to be reported and communicated to the building owner and occupants. The results must be interpreted, and their significance explained in layman’s terms. It is fundamental that the report states the source and cause of moisture, a plan for repair and a risk assessment. The report should also contain all investigation steps with photo documentation, a description of the performed analyses and procedures, analyses’ results and conclusions. Finally, an action plan for the renovation of the building should be provided, including means to remove the fungal growth, a cleaning scheme and quality control of fungal removal.

## 5. Conclusions and Perspectives

Overall, the literature highlights the challenges of investigating the existence of fungal growth. At the same time, a targeted approach is often needed where the inspector knows which species to look for [34,124]. Focusing on specific fungal groups and species likely to grow in damp indoor environments and on the present, specific building materials can help, for example, to choose the suitable medium for isolation or adequate detection method. In addition, research has shown that the production of mycotoxins is species-specific [38] and the substrate and its characteristics influence their production [87]. Therefore, a better understanding of the associations between fungal species and various construction materials can be employed to limit adverse health effects stemming from exposure to building-related fungal species, as well as reduce material decay [16]. Nevertheless, standardised, widely accepted protocols and guidelines are missing [124,125,127,132], making it difficult to obtain reproducible and comparable results, as well as definite recommendations on fungal contamination problems.

This review aims to guide fungal assessment inspections by aiding in selecting the most suitable sampling and detection methods available, predicting the type and location of moisture damage and interpreting the results of the findings. It is essential to establish whether fungal growth is linked to humidity problems or if a hygiene issue is more likely, which can only be done using species identification methods [32].

If the inspector detects one or more fungal species from Table 3, Table 4, Table 5 and Table 6, it suggests that there is a dampness-related fungal contamination problem and proposes which materials to focus on. Researchers can use the tables as a starting point for developing an extended database of the associated fungal species for all building materials. For professionals developing guidelines and standards about indoor microbial contamination, this guide can be useful for bringing up-to-date older publications while indicating which fungal species are likely to be present in buildings. Finally, this work seeks to underline and draw attention to the importance of collaboration between different disciplines (i.e., building specialists, mycologists and health professionals) regarding fungal growth risk assessment in buildings.

## Figures and Tables

**Table 7:** Advantages and limitations of commonly used sampling techniques [18,126,130,131,133,139,146].

		Advantages	Limitations
Material	Bulk	Allows further growth in the lab	Destructive sampling
		Examination of reverse subsurface layers	No standardised procedures
		Can sample a large area	Some materials can be hard to sample (e.g., concrete)
		Several sub-samples can be taken from one sample	
	Scrapings and shavings	Allows further growth in the lab	Destructive sampling
		Examination of reverse subsurface layers	No standardised procedures
		Can sample a large area	Qualitative or semi-qualitative analysis
		Easier collection of some materials than bulk samples (e.g., drywall)	
	Tape lifts	Fast analysis	No standardised procedures
		Collection of viable and non-viable spores	No separation between viable and non-viable spores
		Analysis of a specific surface (targeted approach)	Location-specific (sampling area is important)
		Quality control after remediation	
		Samples can be stored for fairly long periods	
	Contact plates	Detect active surface contamination	Many samples with different medium are required for characterisation of the environment
	Swabs	Easy to collect	
		Suitable for hardly accessed surfaces	
Dust	Swabs	Less susceptible to short-term fluctuations	
	Vacuum cleaner	Easy sample collection	Not widely used
		Can sample a large area	No standardised procedures
		Can be divided into many sub-samples	
	DFC/EDC	Easy sample collection	Dust reserves’ activation for large particles
		No special equipment required	Slow
Air	Passive: Petri dish	Easy sample collection	No quantification
		Can sample a large area	Collection of a small air volume
		No air sampler is required	No standardised procedures
	Impaction: Petri dish	Can be quantified	Air sampler is required
	Impaction: sticky surf.	Easy use	Air sampler is required
		Relatively fast results	No species-level identification
		Can be used to detect moisture-specific genera	No sampling of reproductive structures
			Short, not representative sampling time
	Liquid impinger	Can be divided into many sub-samples	Air sampler is required
		No sample extraction needed	Risk of fluid evaporation and spore diffusion
			Difficult to handle liquid samples and glass impingers in the field
			All liquids have advantages and limitations
	Filter cassettes	High collection efficiency for a wide particle range	Air sampler is required
		Long sampling times	High detection limit for microscopic counting
		Can be divided into many sub-samples	Possible desiccation of sensitive microorganisms
			Extraction procedure can affect the results
	Centrif./ cyclonic	High-volume sampling	Air sampler is required

**Table 8:** Sampling techniques and corresponding detection methods.

		Detection/Identification Methods			
Sampling Techniques		Direct Microscopy	Culture-Based	Molecular Anal. (qPCR)	Enzymatic Anal. (NAHA)
Material	Bulk materials	x			
	Scrapings and shavings	x	x		
	Tape lifts on surface	x			
	Contact plates on surface	x	x		
	Swabs on surface		x	x	x
Dust	Swabs		x	x	x
	Vacuum cleaner		x	x	x
	Sedimentation on DFC ^1^/EDC ^2^		x		
Air	Sedimentation on Petri dish	x	x		
	Impaction on Petri dish	x	x		
	Impaction on adhesive surface	x			
	Impingement in liquid		x	x	x
	Filter sampling		x	x	x
	Centrifugal or cyclonic samplers		x	x	x

^1^ Dust fall collector. ^2^ Electrostatic dust fall collector.

**Table 9:** Advantages and limitations of commercially used analytical methods.

	Advantages	Limitations
Direct microscopy	Fast	Only genus identification
	Both viable and non-viable fungal biomass	Only qualitative
	Distinction between spores and mycelium	Requires a mycologist
Culture-based analysis	Species identification	Slow
	Widely used, well characterised and researched	Only viable biomass
	Large reference data available	Growth is medium- and conditions-dependent
	Semi-quantitative for species	Overgrowth of fast-growing/heavy-sporulating fungi
		Requires a mycologist
Molecular analysis (qPCR)	Fast	Limited to targeted species
	Species identification of building-associated fungi	No detection of yeasts
	Quantitative for species	No detection of fungal structures without a nuclei
	Both viable and non-viable fungal biomass	
	Requires a technician	
Enzymatic analysis (NAHA)	Fast	No fungal identification
	Quantitative for biomass	No detection of yeasts
	Both viable and non-viable fungal biomass	Sensitive to dirt
	Requires a technician	

## Supplementary Materials

The supporting information for the recipes for media can be downloaded at: https://www.mdpi.com/article/10.3390/jof10020108/s1. References [64,65,66] are cited in supplementary materials.
