# Supplementary material for: Damp Buildings: Associated Fungi and How to Find Them
Source: J Fungi (Basel). 2024 Jan 27;10(2):108. doi: 10.3390/jof10020108 (PMC10890273; doi:10.3390/jof10020108)
Supplement: Supplementary file 1 [file jof-10-00108-s001.zip › jof-2806075-supplementary.pdf]

Supplementary material for “Damp buildings: Associated Fungi and How to Find Them.”

Recipes for media from Samson RA, Houbraken J, Thrane U, Frisvad JC, Andersen B. *Food and Indoor Fungi*, 2nd ed; Westerdijk Fungal Biodiversity Institute: Utrecht, The Netherlands, 2019.

For suppression of bacterial growth, especially *Bacillus*, 50 ppm chlortetracycline and 50 ppm chloramphenicol should be added to all media if they are used for direct plating. Only chloramphenicol can withstand autoclaving without loss of activity.

Descriptions are based on the use of yeast extract from Difco and malt extract from Oxoid. All media have $Znso_4$ and $CuSO_4$ supplemented to ensure proper colony/conidium colour. Lack of trace metals prevents the formation of melanin, yielding pale, white or brown colonies, especially in *Penicillium*.

DG18: Dichloran 18 % Glycerol agar	
Dichloran-Glycerol-agar-base (Oxoid)	31.5 g
Glycerol (anhydrous)	220.0 g
$ZnSO_4 \cdot 7H_2O$	0.01 g
$CuSO_4 \cdot 5H_2O$	0.005 g
Chloramphenicol	0.05 g
Distilled water	1000 ml
Mix well and autoclave at 121 °C for 15 min. After autoclaving, add 0.05 g chlortetracycline. pH: 5.6 ± 0.2 (Hocking & Pitt, 1980).	

MEA: Malt Extract Agar	
Malt extract (Oxoid CM0059)	50.0 g
$ZnSO_4 \cdot 7H_2O$	0.01 g
$CuSO_4 \cdot 5H_2O$	0.005 g
Distilled water	1000 ml
Mix well and autoclave at 115 °C for 10 min. pH: 5.4 ±0.2.	

MY50G: Malt Yeast 50 % Glucose agar	
Malt extract (Oxoid CM0059)	10.0 g
Yeast extract (Difco)	2.5 g
$ZnSO_4 \cdot 7H_2O$	0.01 g
$CuSO_4 \cdot 5H_2O$	0.005 g
Glucose	500.0 g
Agar	10 g
Distilled water up till	500 ml
Mix ingredients, except glucose, in water and boil till agar is dissolved. Add glucose while still hot. Steam (100 °C) for 30 min. pH: 5.3 ± 0.2 (Pitt & Hocking, 2009).	

V8: Original V8® Vegetable Juice agar	
V8® vegetable juice (Campbell)	175 ml
$CaCO_3$	3.0 g
$ZnSO_4 \cdot 7H_2O$	0.01 g
$CuSO_4 \cdot 5H_2O$	0.005 g
Agar	20.0 g
Distilled water	825 ml
Mix well and autoclave at 121 °C for 15 min. pH: 6.4 ± 0.1. (Simmons, 2007).	

Stock solutions of trace metals, antibiotics and other minor components can be made in advance and stored at 5 °C in the dark.

References

Hocking AD, Pitt JI (1980). Dichloran-glycerol medium for enumeration of xerophilic fungi from low moisture foods. Applied and Environmental Microbiology 39: 488–492.

Pitt JI, Hocking AD (2009). *Fungi and Food Spoilage*. 3rd ed. Springer: Dordrecht, The Netherlands.

Simmons E.G. (2007). *Alternaria: An Identification Manual*; CBS Fungal Biodiversity Centre: Utrecht, The Netherlands.
